# Silica-Based Monolithic Columns as a Tool in HPLC—An Overview of Application in Analysis of Active Compounds in Biological Samples

**DOI:** 10.3390/molecules25143149

**Published:** 2020-07-09

**Authors:** Michał Staniak, Magdalena Wójciak, Ireneusz Sowa, Katarzyna Tyszczuk-Rotko, Maciej Strzemski, Sławomir Dresler, Wojciech Myśliński

**Affiliations:** 1 Department of Analytical Chemistry, Medical University of Lublin, Chodźki 4a, 20-093 Lublin, Poland; i.sowa@umlub.pl (I.S.); maciej.strzemski@poczta.onet.pl (M.S.); 2 Faculty of Chemistry, Institute of Chemical Sciences, Maria Curie-Skłodowska University Lublin, 20-031 Lublin, Poland; ktyszczuk@poczta.umcs.lublin.pl; 3 Department of Plant Physiology and Biophysics, Maria Curie-Skłodowska University, 20-033 Lublin, Poland; dresler.slawomir@gmail.com; 4 Chair and Department of Internal Diseases, Medical University of Lublin, 20-081 Lublin, Poland; wojciech.myslinski@umlub.pl

**Keywords:** monolithic column, stationary phases in HPLC, drug analysis, plant analysis

## Abstract

Monolithic fillings used in chromatography are of great interest among scientists since the first reports of their synthesis and use were published. In the 20 years since silica-based monolithic columns were introduced into the commercial market, numerous papers describing their chromatographical properties and utility in various branches of industry and scientific investigations were presented. This review is focused on possible applications of commercially available silica-based HPLC monolithic columns in the analysis of biological samples.

## 1. Introduction

High-performance liquid chromatography (HPLC) is a dynamically developing technique widely used in almost all branches of industry and pharmaceutical, chemical, and agri-food investigations, as well as in laboratory practice and scientific research [1]. This technique is based on the separation of target compounds from a matrix of samples containing other accompanying constituents; therefore, the chromatographic column filled with the stationary phase where the separation process takes place is named “the heart of the chromatographic system”. Currently, columns with various types of fillings are commercially available; however, spherical packed columns are still most commonly used.

The historical background of all monolithic columns was brilliantly presented by Svec et al. [2]. Monolithic stationary phases were the subject of interest for many research groups over the last 30 years. They are often called “monolithic rods” [3] or “silica rods” in the case of silica monolithic columns [4]. Due to the characteristic structure that distinguishes them from traditional spherical fillings and their numerous advantages, including low susceptibility to clogging and low flow resistance, they are a very interesting alternative for many scientists [5]. Considering the type of material used for synthesis, monolithic columns can be divided into two groups; the first is based on silica gel and the second is based on polymeric materials [6]. Vyviurska et al. presented an exhaustive comparison of both types of commercially available monoliths [7]. The major disadvantage of most polymeric monolithic fillings is their inability to separate small molecules [8]; hence, their significance in the analysis of samples with a complex matrix such as plant material is low. They are mostly applied for analysis of compounds with high molecular weight such as proteins or polynucleotides [9,10,11,12] and they have greater importance in electrochromatographic techniques [13,14]. Moreover, although polymeric monolithic columns are produced by some manufacturers such as BIA Separations (Ljubljana, Slovenia), Bio-Rad Laboratories (Hercules, CA, USA), or Thermo Scientific (Dionex Corporation) (Sunnyvale, CA, USA) [15], the majority of reports concern home-made fillings and, in these cases, the reproducibility of results is difficult to obtain because the process of synthesis conducted by different researchers may slightly differ [14,16].

Many published studies showed the applicability potential of silica-based monolithic columns in investigations of various samples, including plants [17,18,19,20], food [21,22,23], dietary supplements [24], and drugs [25,26,27]. So far, numerous review papers described the analytical use of monolithic columns [1,4,5,28,29,30,31,32,33,34,35]. Namera et al. showed applications of different types of commercially available silica-based monolithic columns in the analysis of active compounds in biological materials. It is worth noting that the Chromolith^®^ Performance RP-18e column (100 × 4.6 mm) was used most commonly [32]. Maruška et al. presented possible applications of monolithic equipment in phytochemical analysis [20]. Monolithic columns are also widely used in proteomics and metabolomics. Rigobello-Masini et al. presented detailed information on the potential applications of this type of chromatographic filling in this research area [31]. The aim of our study is to summarize and update the possible applications of this type of fillings. The review covers papers published after 2006 and focuses on commercially available columns, as, due to the complexity and diversity of the manufacturing process, the batch-to-batch reproducibility of home-made fillings is poor. Currently, two companies produce monolithic columns based on silica for HPLC—Merck KGaA (Darmstadt, Germany) and Phenomenex (Torrance, CA, USA). Their products are available under trade names Chromolith^®^ and Onyx™, respectively.

## 2. Way of the Silica Monolith to the Commercial Market

Initial work on the synthesis of monolithic silica dates to the early 1990s when Nakanishi and Soga presented the process of continuous silica synthesis with two types of pores [36,37,38]. A patent describing the synthesis of the silica-based monolithic rod was registered in Japan in 1993 and then in the United States in 1997 [3]. In 1996, Tanaka et al. used this synthesized monolithic stationary phase for the first time to separate aromatic hydrocarbons and insulin [39]. In 2000, Merck launched the first generation of commercially available silica-based monolithic columns from the Chromolith^®^ series [40]. This moment was a breakthrough and caused a significant increase in interest in this type of chromatographic filling [3,4]. The monolithic structure of the silica rod from which the first-generation monoliths were built was not perfect and had some limitations, such as the broad size distribution and accidental distribution of through-pores [41]. Moreover, Gritti et al. indicated radial heterogeneity as one of the disadvantages of the first-generation monoliths [42,43]. Changes introduced in the process of the synthesis of a monolithic rod (increased amount of porogen) led to creation of the so-called second-generation monoliths [44]. Better control of the production process and reduced size of macropores meant that the second generation of monoliths had better separation efficiency and better peak symmetry [40]. This resulted in the introduction of commercially available second-generation monoliths under the trade name Chromolith^®^ High Resolution by Merck in 2011. A detailed comparison of the chromatographic and physicochemical features of both generations of monoliths was presented in several papers [45,46,47,48].

## 3. Main Features and Synthesis of the Silica-Based Monolithic Rod 

HPLC silica-based monolithic columns are made from one continuous, rigid piece of porous silica sealed in a polyether ether ketone (PEEK) tube. Numerous advantages of monolithic fillings result from the characteristic morphology of silica gel. This structure is characterized by a bimodal pore size distribution and two types of pores, macropores and mesopores, can be distinguished [3,4]. Macropores, also called “through-pores” [3] or “flow pores” [41], form a network of connections and are responsible for the high permeability of the bed, whereas mesopores provide the surface needed for proper chromatographic separation [49]. The high permeability of the monolithic bed and the low backpressure allow high flow rates of the mobile phase and, thus, a significant reduction of the analysis time while maintaining satisfactory chromatographic separation parameters [50].

The sol–gel process including the hydrolysis reaction and polycondensation of organosilica compounds in the presence of a water-soluble polymer is applied in the process of manufacturing of monolithic silica rods. Tetraalkoxysilanes, i.e., tetramethyl-ortosilane (TMOS) and tetraethyl-ortosilane (TEOS), are silica precursors during the synthesis [51]. Currently, TMOS is the most commonly used alkoxysilane to prepare silica-based HPLC monolithic columns [28]. Nakanishi and Soga were pioneers in the synthesis of monolithic silica rods. In the first paper describing the process of synthesis of a monolithic silica bed, they used a mixture of TMOS and poly(sodium styrene sulfonate) with different molecular weights as a starting solution [36]. Later, this method was modified by replacing poly(sodium styrene sulfonate) with polyacrylic acid with the addition of nitric acid as a hydrolysis catalyst [38]. Subsequent modifications consisted of a change in the molecular weight of polyacrylic acid [37] and, next, the introduction of polyethylene oxide [39,52]. Subsequent synthesis steps include aging, drying, and chemical modification of the obtained gel. Modifications at each of these stages cause changes in the morphology of the monolithic silica. An increase in the TMOS concentration in the starting solution increases the mechanical strength of the monolith. The most popular additive used in the synthesis is polyethylene glycol. Both the concentration and the molecular weight of the additives used in the starting solution have an impact on the morphology of the gel. An increase in the polyethylene glycol concentration causes a decrease in the size of the through-pores [28]. The characteristics and detailed description of the individual stages of the synthesis were comprehensively presented in reviews by Guichon [3] and Rieux et al. [28].

## 4. Applicability of the Monolithic Column

HPLC equipped with reverse-phase spherical packed columns is commonly used to identify and evaluate the content of active compounds in plant- and human-derived material. The multitude of commercial products available on the market and the diverse properties of the fillings allow choosing a column dedicated to particular types of analytes. However, the main problem of biological samples is their rich matrix, which may cause clogging of adsorbent pores. This in turn decreases the separation efficacy and, consequently, shortens the longevity of the bed. The unique structure of the monolithic column can partly solve such problems; therefore, the application potential of monolithic beds was intensively studied over the last 20 years. Monolithic analytical HPLC columns with different lengths (100, 50, 25 mm) and different internal diameters (2, 3, 4.6 mm) are currently available on the commercial market. Short columns allow ultra-fast separation of samples with a relatively simple matrix, while longer columns facilitate analysis of much more complex mixtures. The most popular and the most frequently used column from the Merck Chromolith^®^ series is Chromolith^®^ Performance RP 18e 100 × 4.6 mm. Some authors did not provide the full trade name of the column used. During our research, we found numerous descriptions such as “Chromolith^™^ RP-18e”, “Monolithic RP-18e”, “Chromolith RP-18e”, “Chromolith C18”, and “Chromolith RP-18”. To the best of our knowledge, all these descriptions refer to the same column.

### 4.1. Plant Samples

The analysis of plant material is very difficult because of the multitude of components often hindering the proper separation analytes from accompanying compounds. However, there are quite many reports describing the application of monolithic columns for the determination of compounds from various chemical groups, such as flavonoids [53,54,55,56,57,58,59], phenolic acids [59,60,61,62], alkaloids [63,64,65,66], furocoumarins [67], and saponins [19]. It can be observed that the efficacy of separation of particular groups of analytes strongly depended on the chromatographic conditions used. For instance, Biesaga et al. [54] obtained full separation of six flavonoids, including quercetin and naringenin, using isocratic elution with 50 mM phosphate buffer and acetonitrile (75:25, *v*/*v*). In turn, the resolution of these compounds in the chromatographic conditions proposed by Repollés et al. [55] was poor. Good resolution between quercetin, miricetin, and kampferol at a flow rate increased to 4 mL/min was obtained for an eluent composed of acetonitrile and orthophosphate buffer (38:62, *v*/*v*), which ensured shortening of the analysis time from 11 min to 60 s [58], compared with the aforementioned papers. Generally, different conditions of separation were proposed for the same group of analytes, taking into account the accompanying matrix. For polyphenols in apple peel extract, Chinnici et al. [59] developed a gradient elution program using 0.5% methanol in 0.01 M phosphoric acid and acetonitrile at a flow rate of 2.5 mL/min and a temperature of 25 °C. In turn, a similar group of compounds in grapes were analyzed using gradient elution with water and methanol acidified with acetic acid [60]. On the other hand, isocratic elution with the mobile phase consisting of acetonitrile and 0.05% trifluoroacetic acid (12:88, *v*/*v*) at an increased flow rate (4 mL/min) and temperature (35 °C) ensured good separation of polyphenols in *Vanilla planifolia* extract. Generally, evaluation of the chromatographic performance systems reported in the literature is very difficult because no detailed chromatographic parameters were shown. In most papers, only retention times of investigated analytes were given, and some authors included the resolution (R_S_) between neighboring peaks as the most important factor from the point of view of the applicability of monolithic columns. Table 1 summarizes the chromatographic conditions used in the analysis of particular classes of compounds in various plant samples.

The main advantage of monolithic fillings is the high permeability of the bed and the low backpressure generated on the column, which makes it possible to use high mobile phase flow rates without loss of separation efficacy. The mobile phase composition used in monolithic fillings is typical for the HPLC technique; however, many chromatographic separations are conducted using a higher flow rate, even up to 6 mL/min, which results in shortening of the time necessary for the chromatographic run. Some authors compared the capabilities of monoliths and columns with spherical filling. Barbero et al. separated five capsaicinoids from hot peppers using a mobile phase flow rate of 6 mL/min, which significantly reduced the analysis time compared to analysis carried out with the use of traditional fillings [68]. Sharma et al. applied a flow rate of 4 mL/min and presented the separation of four components from vanilla extracts in less than 3 min. The authors compared the developed method for the monolith with UPLC. Most of the chromatographic parameters (except the theoretical plate number) were better using the HPLC method with a monolithic bed, whereas the UPLC method was characterized by lower consumption of the mobile phase and higher sensitivity [61]. Using a flow rate of 4 mL/min, Mehrad et al. specified the conditions for separation of three major flavonol aglycones from *Rhus coriaria*. The method yielded good resolution of the analyzed compounds in less than 1 min [58]. Pellati et al. analyzed polyacetylenes and polyenes from *Echinacea pallida* roots using a monolithic bed and obtained shorter retention times and better separation of the analytes than in the case of a spherical packed column [17]. Rahim et al. presented simultaneous determination of eight catechins and caffeine in tea samples on the monolithic stationary phase. The authors highlighted the short time of analysis as the main advantage of their methodology [63]. The influence of the increased flow rate on resolution parameters was also studied. Liazid et al. [60] investigated the separation of polyphenolic compounds using different flow rates of the mobile phase in the range of 2–5 mL/min. Generally, a slight decrease in the Rs values was observed, but some compounds co-eluted at a higher flow rate.

An interesting alternative is the possibility of connecting several monolithic columns, which allows lengthening the separation way and, hence, increasing the efficacy of separation. For example, Schmidt developed a fast method for quality control of *Harpagophytum procumbens* using two coupled monolithic columns at a flow rate of 5 mL/min. This method contributed to shortening the analysis time by almost 25 min in comparison with the use of a spherical packed column. The same method was successfully used to distinguish between the *Harpagophytum* species [69,70].

Another application of the monolithic column is fingerprinting, which is accepted by the World Health Organization (WHO) as a valuable tool for assessing the quality of plant samples [71]. Alaerts et al. coupled four monolithic columns together and obtained chromatographic fingerprints for four *Artemisia* species [72], whereas Hefny Gad et al. used two combined monoliths to obtain HPLC fingerprints of *Ipomea aquatica* samples [73].

### 4.2. Medical and Pharmaceutical Application

Over the last few years, monolithic columns were also widely used in the analysis of drugs and their metabolites in various matrices. Some reports presented the usefulness of monoliths in the analysis of urine, saliva [74,75], whole blood [76], plasma [75,77], serum, and human breast milk samples [78]. The papers described the chromatographic analysis of compounds from different therapeutic groups, such as antibiotics [25,77,79,80], diuretics [75,77,81], antidepressants [82,83], analgesics [74], antidiabetics [84,85], benzodiazepine derivatives [76,86,87], and anti-allergic [88] and antiviral drugs [26,89]. Table 2 presents examples of applications of monolithic columns in medical analysis.

For instance, Galaon et al. [77] and Wenk et al. [75] developed very fast methods for determining furosemide in human plasma, which can be useful in bioequivalence and pharmacokinetic studies. In the conditions reported in the papers, furosemide was eluted at approximately 4 and 2 min, respectively; however, the eluent proposed by Galaon et al. [77] had more components, including sodium heptane-sulfonate, trimethylamine, methanol, and acetonitrile, while retention was based on the ion-pair mechanism. In turn, Wenk et al. [75] used gradient elution with a simple mobile phase containing acetonitrile and water with the addition of acetic acid. 

Three different chromatographic systems including various eluent compositions and flow rate values were designed for determination of similar analytes from the benzodiazepine group in human blood samples. In all cases, the obtained retention times were similar and the compounds were well separated from the matrix [76,86,90]. Karageorgou et al. presented the first method for separation and quantification of residues of eight cephalosporins in milk in a shorter time than 16 min. The use of the monolithic column ensured a significantly shorter total analysis time than other analytical columns (22 min for Inertsil ODS-3 5 μm, 250 × 4 mm and 43 min for Orbit 100 C_18_ 5 μm, 250 × 4 mm); hence, the consumption of eluents was lower [80]. Ardakani et al. described a rapid method for determination of tramadol and its main metabolites with the use of simple isocratic elution without conditioning of the bed between injections [74]. The total analysis time was about 7 min in the case of the monolithic column and about 26.5 min for the traditional column [91].

Monolithic filings also help to avoid the time-consuming preparation of the sample for chromatographic analysis. Bugey et al. developed a semi-automatic method to analyze benzodiazepines in whole blood samples, in which two monolithic columns of different lengths and various purposes were switched together. The first one (Chromolith^®^ Flash, 25 × 4.6 mm) was used for sample clean-up, while the other (Chromolith^®^ Performance RP-18e, 100 × 4.6 mm) served as an analytical column. This solution ensured purification and proper separation during one injection; hence, the whole analytical process was substantially shortened [76].

### 4.3. New Generation of Monolithic Columns—Short Characterization and Applications

Chromolith^®^ High Resolution is an example of second-generation monolithic columns, which were introduced on the market in 2011 [44]. The main goal in the development of the manufacturing process of second-generation monoliths was to enhance the separation efficiency and decrease the peak asymmetry [40]. The reduced size of macropores and the more homogeneous structure of the high-resolution monolithic rod are the main features distinguishing both generations [40,46]. Changes in the morphology of monolithic silica contributed to improving column efficiency by reducing the height of the individual theoretical plate [48]. The second generation of monoliths shows performance similar to the sub-3-μm core shell and sub-2-μm fully porous particles [92]. Changes in the morphology of second-generation monoliths resulted in improvement of the chromatographic parameters and an increase in the efficacy of separation; however, an increase in the backpressure compared to the first generation was observed [40]. Although the permeability of the second-generation monoliths is even four times lower than in the first generation [44], the backpressure during analysis is still much lower than in a particle packed column.

Table 3 summarizes the applications of the Chromolith^®^ High Resolution (100 × 4.6 mm) column. Most of them are associated with the analysis of drugs in biological fluids. Kučerová et al. proposed a very interesting comparison of the second generation of monoliths, as well as core–shell and particle packed columns, in the analysis of retinol and α-tocopherol in various matrices using the UHPLC system. It was found that the High Resolution monoliths were comparable and, in some cases, even better than the other UHPLC columns [78]. Koyuturk et al. presented a method where they used a double gradient, i.e., both the mobile phase and the flow rate, for simultaneous determination of irbesartan and hydrochlorothiazide in urine samples. The method developed with the use of Chromolith^®^ High Resolution yielded the highest theoretical plate number, compared with other tested columns (with dimensions 100 × 4.6 mm) [81].

### 4.4. Preparative and Semi-Preparative Silica-Based Monolithic Column—Applications

Raw materials of plant origin are a valuable source of active compounds widely used in many fields, e.g., natural medicine and pharmaceutical and herbal industries. Some species are a valuable source of biological active compounds with desirable therapeutic effects useful for treatment of various disorders. The preparative and semi-preparative chromatography technique allows purification of raw extracts from ballast substances and separation into particular fractions rich in compounds with similar chemical and biological properties. In the case of chromatographic columns used on a preparative and semi-preparative scale, a crucial point is the possibility of loading a high volume of a sample, conducting the separation process without the risk of overloading the entire system. Silica-based monolithic columns for preparative and semi-preparative purposes with dimensions 100 × 25 mm and 100 × 10 mm, respectively, are offered by Merck (Darmstadt, Germany). There are several examples of the use of this type of column in purification and isolation processes [93,94,95,96]. For instance, Lai et al. applied a monolith for isolation of proanthocyanidin from *Blechnum orientale* [96], and Malek et al. used a monolithic column for preparative scale separation of active components from *Curcuma manga* [94]. A combination of a few columns was also applied for preparative purposes, e.g., Kokotkiewicz et al. used two semi-preparative Chromolith^®^ columns connected in series for the isolation of phenolic compounds from *Cyclopia subternata* [97].

## 5. Conclusions

As shown in our study, columns with monolithic beds have great application potential for a wide range of analytes, including polar and low-polarity compounds from various chemical groups. Moreover, due to their unique pore structures, which result in numerous advantages such as low backpressure at high flow rates of eluents and low susceptibility to clogging, they are a useful tool in the analysis of samples with a rich matrix, including plant material and human-derived samples. Many chromatographic conditions, including various compositions of the mobile phase, flow rate values, temperatures, and types of elution were elaborated, taking into account the type of samples and analytes. Additionally, the combination of a few columns enhances the separation effectiveness of monoliths. The possibility of applying an increased flow rate of eluents allows shortening the time of analysis. Columns with monolithic filling also have increasing significance for preparative and semi-preparative applications, and they are used for isolation and purification of target compounds. The new generation of monolithic columns with improved efficiency of separation can increase their importance in chromatography in the future.

## Figures and Tables

**Table 1:** Application of Chromolith^®^ Performance RP 18-e (100 × 4.6 mm inner diameter (i.d.)) in analysis of plant samples.

Sample/Analytes	Part of Plant/Matrix	Type of Elution/Mobile Phase	Conditions (Flow Rate/Temperature/Numbero f Monolithic Columns)	Detector	Ref.
orientin, isovitexin, vitexin, luteolin-7-*O*-glucoside, hyperoside, luteolin, apigenin	tincture from *Passiflora incarnata* L.	gradient elution/ H_2_O/MeOH ^a^/ACN ^b^/THF ^c^ acidified with 0.05% acetic acid	1.0 mL/min/30 °C/one column 2.0 mL/min/30 °C/two columns 2.5 mL/min/30 °C/three columns	PDA	[53]
polyacetylenes and polyenes	roots from *Echinacea pallida*	gradient elution/ H_2_O/ACN	2.0 mL/min/20 °C/one column	PDA	[17]
(fingerprinting)	*Artemisia vulgaris*, *A. absinthium*, *A. annua*, *A. capillaris*	gradient elution/ H_2_O/MeOH (both with 0.05% of TFA ^d^)	1.0 mL/min/35 °C/four columns	DAD	[72]
catechins and caffeine	tea samples (green tea, Oolong tea, “fermented” black tea)	isocratic elution/ H_2_O/ACN/MeOH (83:6:11, *v*/*v*)	1.4 mL/min/-/one column	UV	[63]
quercetin, naringenin, naringin, myricetin, rutin, kaempferol	tomatoes	isocratic elution/ A: 50 mM phosphate buffer (pH = 2.2)/ACN (75:25, *v*/*v*) B: 2 mM formic acid/ACN (75:25, *v*/*v*)	1.0 mL/min/25 °C/one column	A: UV B: MS	[54]
gallic acid, protocatechuic aldehyde, gentisic acid, catechin, vanillinic acid, caffeic acid, vanillin, epicatechin, syringaldehyde, p-coumaric acid, ferulic acid, sinapic acid, resveratrol	musts from grapes: Riesling and Monastrell	gradient elution/ 90% H_2_O, 2% acetic acid in MeOH/90% MeOH, 2% acetic acid in H_2_O	2.5 mL/min/25 °C/one column	PDA and FL	[60]
catechin, epicatechin, quercetin, kaempferol, apigenin, fisetin, morin, naringenin, hesperetin, chrysin	green tea, red wine, orange, propolis and *Ginkgo biloba* extracts	gradient elution/ H_2_O/MeOH/ACN each containing 0.05% (*v*/*v*) TFA	2.0 mL/min/25 °C/one column	DAD	[55]
(fingerprinting)	aerial parts from *Ipomoea aquatica*	gradient elution/ MeOH/H_2_O containing 0.05% TFA	1.0 mL/min/25 °C/two columns	UV	[73]
gastrodin	*Gastrodiae Rhizoma*	gradient elution/H_2_O/ACN	1.0 mL/min/-/one column	DAD	[18]
echitamine, N-demethylalstogustine, loganetin	stem, stem bark, root, root bark, fruits, leaves from *Alstonia scholaris*	isocratic elution/ ACN/0.01 M buffer (KH_2_PO_4_) containing 0.1% TFA (20:80, *v*/*v*)	0.5 mL/min/25 °C/two columns (total length 150 mm)	DAD	[98]
oroxylin A, chrysin, baicalein, hispidulin	roots from *Oroxylum indicum*	isocratic elution/ACN/H_2_O (acidified with 0.1% TFA) (34:66, *v*/*v*)	1.0 mL/min/30 °C/one column	PDA	[56]
6-gingerol, 8-gingerol, 10-gingerol, shogaol	rhizome from *Zingiber officinale*	gradient elution/ H_2_O/ACN	3.0 mL/min/room temp./one column	PDA	[99]
schizandrin, gomisin A, deoxyschizandrin, γ-schizandrin, gomisin N, wuweizisu C	callus from *Schisandra chinensis*	isocratic elution/ ACN/H_2_O (50:50, *v*/*v*)	2.0 mL/min/-/one column	PDA	[100]
bacopaside I, bacoside A_3_, bacopaside II, bacopaside X, bacopasaponin C, apigenin	herbs of *Bacopa monnieri*	isocratic elution/ ACN/H_2_O (30:70, *v/v*)	0.7 mL/min/25 °C/one column	ELSD	[19]
vanillin, vanillic acid, *p*-hydroxybenzoic acid, *p*-hydroxybenzaldehyde	pods from *Vanilla planifolia*	isocratic elution/ ACN/0.05% TFA in H_2_O (12:88, *v*/*v*)	4.0 mL/min/35 °C/one column	PDA	[61]
furocoumarins: heraclenol and bergapten	fruits from *Heracleum candicans*	gradient elution/ H_2_O/H_3_PO_4_ (99.7:0.3, *v*/*v*)/ ACN/H_2_O/H_3_PO_4_ (79.7:20:0.3, *v*/*v*)	0.5 mL/min/-/one column	PDA	[67]
tannins and polyphenols	commercial products *Filipendula ulmaria* *Rosa canina*	gradient elution/ACN/H_2_O containing 0.2% (*v*/*v*) formic acid	2.5 mL/min/-/one column	UV	[101]
phenolic acids: vanillic, gallic, syringic, *p*-coumaric, ferulic, chlorogenic, benzoic, *p*-hydroxybenzoic, *p*-hydroxyphenylacetic	plum fruits	gradient elution/ 50 mM phosphate buffer (pH = 2.2)/ACN	1.0 mL/min/-/one column	DAD	[62]
niaziridin and niazirin	leaves, pods, and bark from *Moringa oleifera*	isocratic elution/ MeOH/sodium dihydrogen phosphate–acetic acid buffer (0.1 M, pH = 3.8) (20:80, *v*/*v*)	0.7 mL/min/25 °C/one column	PDA	[102]
A. fatty acid methyl esters B. phosphatydylocholine	---	isocratic elution/ A. ACN/H_2_O (97:3, *v*/*v*) B. ACN/MeOH/H_2_O (33:64.5:2, *v*/*v*/*v*)	2.0 mL/min/25 °C/two columns	A. radioisotope detector B. UV	[103]
iridoid glycosides: harpagoside and 8-*p*-coumaroyl-harpagide	extracts from *Harpagophytum procumbens* and *H. zeyheri*	gradient elution/ H_2_O (pH = 2.0)/ACN	5.0 mL/min/30 °C/two columns	PDA	[70]
harpagoside, acetoside, cinnamic acid, 8-*p*-coumaroyl-harpagide	root tubers from *H. procumbens*	gradient elution/ H_2_O (pH = 2.0)/ACN	5.0 mL/min/30 °C/two columns	PDA	[69]
curcuminoids: curcumin, demethoxycurcumin, bisdemethoxy curcumin	herbal medicament	isocratic elution/ H_2_O/ACN/glacial acetic acid (60:40:1, *v*/*v*/*v*)	1.0 mL/min/-/one column	UV–Vis	[104]
rutin	Buckwheat Tea and seeds from *Fagopyrum tataricum*	isocratic elution/MeOH/H_2_O (5:5, *v*/*v*) with 10 mM acetate buffer at pH = 4.1	1.5 mL/min/30 °C/one column	UV–Vis	[57]
glycyrrhizic and glycyrrhetinic acids	roots from *Glycyrrhiza glabra*	gradient elution/H_2_O/ACN both acidified with 0.05% TFA	2.5 mL/min/room temp./one column	PDA	[105]
reserpine, ajmaline, ajmalicine	roots from *Rauvolfia serpentina*	gradient elution/ 0.01 M phosphate buffer containing 0.5% glacial acetic acid (pH = 3.5)/ACN	1.0 mL/min/26 °C/one column	PDA	[106]
myricetin, quercetin, kaempferol	fruits and leaves from *Rhus coriaria*	isocratic elution/ACN/10 mM potassium dihydrogen orthophosphate buffer (pH = 3.0) (38:62, *v*/*v*)	4.0 mL/min/40 °C/one column	PDA	[58]
allosecurinine, securinine	biomasses from *Phyllanthus glaucus*	gradient elution/H_2_O/ACN	1.0 mL/min/25 °C/one column	PDA	[64]
proanthocyanidins	pea from *Pisum sativum,* lentil from *Lens culinaris,*faba bean from *Vicia faba*	gradient elution/ H_2_O/ACN both with 1% acetic acid (*v*/*v*)	3.0 mL/min/30 °C/two columns	DAD	[107]
gallic acid, (+)-catechin, chlorogenic acid, procyanidin B2, *p*-coumaric acid, (-)-epicatechin, ferulic acid, hyperin, rutin, phloridzin	fresh peel or pulp from *Golden Delicious* apples	gradient elution/ 0.5% MeOH in 0.01 M H_3_PO_4_/ACN	2.5 mL/min/25 °C/one column	PDA	[59]
capsaicinoids: nordihydrocapsaicin, capsaicin, dihydrocapsaicin, homocapsaicin, homodihydro-capsaicin	peppers (pericarp and placenta) from *Capsicum frutescens*	gradient elution/ H_2_O/MeOH both with 0.1% acetic acid	6.0 mL/min/30 °C/one column	FL	[68]
anthocyanins	red cabbage *Brassica oleracea*	gradient elution/ 5% formic acid/ACN	4.0 mL/min/27 °C/one column	DAD	[108]
protopine, allocryptopine, berberine, chelidonine, chelerythrine, sanguinarine, coptisine	roots from *Chelidonium majus*	gradient elution/ 15 mM ammonium acetate (pH = 4.0)/ACN/MeOH	2.0 mL/min/25 °C/three columns	DAD	[65]
vincristine, vinblastine, catharanthine, vindoline	leaves from *Catharanthus roseus*	isocratic elution/ACN/0.1 M phosphate buffer containing 0.5% glacial acetic acid (pH = 3.5), (21:79, *v*/*v*)	1.2 mL/min/25 °C/one column	PDA	[66]
gallic acid, protocatechuic acid, gentisic acid, chlorogenic acid, caffeic acid, ferulic acid, rosmarinic acid	aerial part from *Hyssopus officinalis*	gradient elution/ H_2_O with 1% acetic acid/ACN	2.0 mL/min/26 °C/one column	DAD	[109]
proanthocyanidins cleavage products	hop cones from *Humulus lupulus* and grapes from *Vitis vinifera*	gradient elution/H_2_O/ACN (each containing 1% acetic acid)	3.0 mL/min/30 °C/two columns	DAD	[110]
daidzin, gycitin, genistin, acetyldaidzin, acetylglycitin, daidzein, glycitein, acetylgenistin, genistein	extracts from *Gycine max*	gradient elution/ ACN/H_2_O with acetic acid (0.1:0.99, *v*/*v*)	flow gradient 3.0 mL and 4.0 mL/min/ two columns	DAD MS	[111]
α-amyrin, α -amyrin acetate, β-amyrin, β-amyrin acetate, lupeol, lupeol acetate	flowers, leaves, roots and stems from five species of *Carlina*	isocratic elution/ACN/H_2_O (95:5, *v*/*v*)	2.0 mL/min/25 °C/one column	PDA	[112]
daidzin, glycitin, genistin, malonyl daidzin, malonyl glycitin, malonyl genistin, daidzein, glycitein, genistein	extracts from soybeans	gradient elution/MeOH/H_2_O each containing 0.1% acetic acid	0.8 mL/min/-/two columns	PDA	[113]
*cis*-resveratrol, *trans*-resveratrol, *cis*-piceid, *trans*-piceid	wine samples	gradient elution/ H_2_O/acetic acid (94:6, *v*/*v*) /H_2_O/ACN/acetic acid (65:30:5, *v*/*v*/*v*)	gradient flow 4.0 mL and 7.0 mL/min/two columns	PDA	[114]
lysergol and chanoclavine	seeds from *Ipomea muricata*	isocratic elution/ ACN/0.01 M sodium dihydrogen phosphate buffer (with 0.2% TFA) (pH = 2.5) (15:85, *v*/*v*)	1.0 mL/min/25 °C/one column	PDA	[115]
rutin, isorhamnetine-3-*O*-rutinoside, isorhamnetine-3-*O*-glukoside, quercetin, isorhamnetin	berries from *Hippophaë rhamnoides*	gradient elution/H_2_O/ACN (both acidified with 1% acetic acid)	3.0 mL/min/40 °C/one column	UV	[116]
geraniin, ellagic acid, gallic acid	rind from *Nephelium lappaceum*	isocratic elution/ACN/H_2_O (30:70, *v*/*v*)	0.5 mL/min/room temp./one column	UV–Vis	[117]
(fingerprinting)	*Ginkgo biloba* dry extract	gradient elution/ *iso*-propanol/THF/H_2_O with 0.05% TFA	1.0 mL/min/35 °C/two columns	UV–ELS	[118]
carnosic acid, carnosol, rosmarinic acid	leaves from *Rosmarinus officinalis*	binary gradient/ACN–H_2_O–H_3_PO_4_ (65.1%:34.9%:0.02%)/ACN–H_2_O–H_3_PO_4_ (22%:78%:0.25%)	1.5 mL/min/-/one column	UV–Vis	[119]
α-solanine and α-chaconine	potato tubers	isocratic elution/20 mM phosphate buffer (pH = 7.8)/ACN (65:35, *v*/*v*)	0.6 mL/min/-/one column	CL	[120]

^a^ MeOH- methanol; ^b^ ACN- acetonitrile; ^c^ THF- tetrachedrofurane; ^d^ TFA- trifluoroacetic acid.

**Table 2:** Chromolith^®^ Performance RP 18-e 100 × 4.6 mm in analysis of drugs in various matrices.

Name of Drug	Matrix	Type of Elution/Mobile Phase	Conditions (Flow Rate/Temperature/Number of Monolithic Columns)	Detector	Ref.
raltegravir	human plasma	isocratic elution/10 mM ammonium formate in water (pH = 3.0)/ACN ^b^ (3:7, *v*/*v*)	1.2 mL/min/40 °C/one column	MS/MS	[89]
amphotericin B	human plasma	gradient elution/ 5 mM ammonium acetate (pH = 6.0)/ACN/MeOH ^a^	1.8 mL/min/-/one column	MS/MS	[25]
lamivudine	human plasma	isocratic elution/ 50 mM sodium dihydrogen phosphate/triethylamine (pH = 3.2) (996:4, *v*/*v*)	1.5 mL/min/20 °C/one column	UV	[26]
mirtazapine and metabolites: *N*-desmethyl mirtazapine, 8-hydroxymirtazapine	human plasma	isocratic elution/ ACN/0.025 M monobasic potassium phosphate buffer (pH = 3.0) (20:80, *v*/*v*)	2.0 mL/min/-/one column	FL	[83]
montelukast and fexofenadine	human plasma	isocratic elution/ 20 mM ammonium formate/ACN (20:80, *v*/*v*)	1.2 mL/min/5 °C/one column	MS/MS	[88]
clonazepam, diazepam, flunitrazepam, lorazepam, midazolam, *N*-desalkylflurazepam, nordiazepam, oxazepam	whole blood samples	isocratic elution/ 5 mM ammonium formate (pH = 3.0)/ACN (65:35, *v*/*v*)	1.5 mL/min/-/one column	MS	[76]
furosemide and norfloxacin	human plasma	isocratic elution/ 0.015 M sodium heptane-sulfonate, 0.2% triethylamine (pH = 2.5)/ ACN/MeOH (70:15:15, *v*/*v*/*v*)	3.0 mL/min/25 °C/one column	FL	[77]
omeprazole	human plasma	isocratic elution/ 0.01 M disodium hydrogen phosphate buffer/ACN (pH = 7.1) (93:7, *v*/*v*),	1.5 mL/min/-/one column	UV	[121]
cefadroxil, cefaclor, cephalexin, cefotaxime, cefazolin, cefuroxime, cefoperazone and ceftiofur	milk	gradient elution/ 0.1% formic acid/ MeOH/ACN (75:25 *v*/*v*)	1.5 mL/min/-/one column	PDA	[80]
pantoprazole	human plasma	isocratic elution/ ACN/potassium dihydrogen phosphate buffer (pH = 3.0) (25:75, *v*/*v*)	1.5 mL/min/-/one column	UV	[122]
codeine	human plasma	isocratic elution/ ACN/10 mM acetic acid (pH = 3.5) (50:50, *v*/*v*)	1.0 mL/min/25 °C/one column	MS/MS	[123]
pioglitazone	human serum and urine	isocratic elution/ ACN/10 mM phosphate buffer (pH = 2.5) (30:70, *v*/*v*)	2.0 mL/min/-/one column	DAD	[124]
diazepam, clonazepam, lorazepam, midazolam	whole blood	isocratic elution/ phosphate buffer (pH = 2.5)/ACN (65/35, *v*/*v*)	2.0 mL/min/-/one column	DAD	[90]
nimesulid and major metabolite 4′-hydroxy-nimesulide	human plasma	isocratic elution/ 0.2% triethylamine (pH = 3.0)/MeOH (50:50, *v*/*v*)	1.5 mL/min/25 °C/one column	DAD	[125]
chloramphenicol	human blood	isocratic elution/ 100 mM phosphate buffer (pH = 2.5)/ACN (75:25, *v*/*v*)	1.5 mL/min/28 °C/one column	UV–Vis	[126]

^a^ MeOH- methanol; ^b^ ACN- acetonitrile.

**Table 3:** Chromolith^®^ High Resolution RP 18-e 100 × 4.6 mm in analysis of active compounds in various matrices.

Active Compound/Drug	Matrix	Type of Elution/Mobile Phase	Conditions (Flow Rate/Temperature/Number of Monolithic Columns)	Detector	Ref.
retinol and α-tocopherol	serum and human breast milk	100% MeOH ^a^	1.5 mL/min/50 °C/one column	FL	[78]
terpenoids and flavonoid aglycones	aerial parts from *Lippia origanoides*	gradient elution/H2O/MeOH both containing 0.1% (*v*/*v*) formic acid	1.0 mL/min/32 °C/one column	UV	[127]
rutin, piceatannol, resveratrol, naringenin, kaempferol, emodin, physcion	root, stem and leaf from five species of *Rumex* L.	gradient elution/ H2O (0.1% formic acid)/ACN ^b^	0.4 mL/min/room temp./one column	MS	[128]
avanafil and its degradation products	pharmaceutical preparation	isocratic elution/H_2_O/ACN both with 0.1% formic acid (pH = 2.6,75:25, *v*/*v*)	0.5 mL/min/40 °C and 15 °C/one column	DAD, MS/MS	[129]
vitamins K3, D3, E, and A	capsules and pediatric drops	isocratic elution/ACN/MeOH both with 0.1% (*v*/*v*) formic acid (pH = 2.6, 25:75, *v*/*v*)	4.0 mL/min/room temp./one column	DAD	[130]
metformin, linagliptin, sitagliptin, vildagliptin	human plasma	isocratic elution/ 0.01 M ammonium formate buffer (pH = 3.0)/ACN (80:20, *v*/*v*)	0.4 mL/min/20 °C/one column	MS/MS	[85]
aspirin and dipyridamole	human plasma	isocratic elution/MeOH/0.1% formic acid in H_2_O (90:10, *v*/*v*)	1.0 mL/min/-/one column	MS/MS	[131]
irbesartan and hydrochlorothiazide	tablets and urine	gradient elution/ ACN/0.025 M phosphate buffer (pH = 6.3)/H_2_O (3:87:10, *v*/*v*)	flow gradient: 0.8 mL and 1.5 mL/min/40 °C/one column	DAD	[81]
dapsone and *N*-acetyl dapsone	human plasma	isocratic elution/ACN/2 mM ammonium acetate in H_2_O (90:10, *v/v*)	0.8 mL/min/-/one column	MS/MS	[132]

^a^ MeOH- methanol; ^b^ ACN- acetonitrile.
